# The Impact of Maternal Gut Dysbiosis on Embryo/Fetus Development

**DOI:** 10.1111/cpr.70124

**Published:** 2025-09-01

**Authors:** Nairui Fan, Yao Shen, Xuesong Yang, Shuxia Ma, Guang Wang

**Affiliations:** ^1^ Basic Medical College of Jiamusi University Heilongjiang China; ^2^ International Joint Laboratory for Embryonic Development & Prenatal Medicine, Division of Histology and Embryology, School of Medicine Jinan University Guangzhou China; ^3^ Key Laboratory for Regenerative Medicine of the Ministry of Education Jinan University Guangzhou China; ^4^ International School Guangzhou Huali College Guangzhou China; ^5^ Guangdong‐Hong Kong Metabolism & Reproduction Joint Laboratory, Guangdong Second Provincial General Hospital, School of Medicine Jinan University Guangzhou China

To the Editor,

1

The gut microbiota is a crucial component of the human body and the most abundant phyla are *Firmicutes* and *Bacteroidetes* [1]. Gut microbiota plays a crucial role in maintaining normal physiological functions of the human body, such as intestinal barrier integrity, immune regulation, and nutrient provision. According to the developmental origins of health and disease (DOHaD) theory, maternal gut dysbiosis during pregnancy may even influence offspring health by altering microbial composition and metabolite profiles. As a key metabolite of the gut microbiota, short‐chain fatty acids (SCFAs) regulate fetal growth and development by influencing transport proteins and widely distributed signalling receptors [2]. In this context, we will focus on the impact of SCFAs on fetal development within the maternal‐fetal environment. Moreover, the increased inflammatory response, oxidative stress imbalance, and changes in metabolites caused by maternal gut microbiota dysbiosis during pregnancy are closely related to the development of the embryonic and fetal cardiovascular system, nervous system, and other systems. Thus, understanding the cellular and molecular biological mechanisms by which maternal gut microbiota and its metabolites regulate embryonic and fetal development will help us improve population health in the future.

The cardiovascular system is the first to emerge during embryonic development, a process regulated by classical developmental signalling pathways such as Wnt, BMP, and Notch [3]. Clinical studies have shown that reduced diversity in the maternal gut microbiota is closely linked to embryonic and fetal cardiac developmental abnormalities. Maternal exposure to selective serotonin reuptake inhibitors (SSRIs) during pregnancy may interfere with serotonin signalling and ultimately affect the gut microbiota. Recent studies have shown that SSRIs can cross the placental barrier and directly influence fetal cardiovascular development [4]. Therefore, the impact of SSRIs on fetal development may involve not direct pharmacological effects but also indirect pathways mediated by the maternal gut microbiome. Current research on the impact of maternal gut microbiota during pregnancy on embryonic/fetal cardiovascular development has focused on the effects of increased lipopolysaccharide (LPS) and decreased SCFA. In mouse experiments, gut dysbiosis increases LPS levels and limits the proliferation, differentiation, and migration of cardiomyocytes. This restriction leads to increased apoptosis and oxidative stress, resulting in a higher incidence of cardiovascular malformations and cardiac bifid as in embryonic and fetal. Moreover, a reduction of SCFAs during maternal pregnancy leads to impaired embryonic angiogenesis and mediates abnormal development of the sympathetic nervous system through GPR41 signalling. Although animal models provide important insights into the role of SCFAs in embryonic cardiovascular development, physiological differences between humans and animals limit the direct clinical applicability of these findings. Further studies are needed to elucidate the molecular mechanisms, gather clinical evidence, and assess ethical feasibility.

The central nervous system (CNS) takes shape during the embryonic development of the neural ganglia. The CNS and the gastrointestinal tract share a bidirectional relationship, known as the gut‐brain axis. Therefore, gut microbiota homeostasis plays a crucial role in the normal growth and development of the embryonic CNS. In mouse models, maternal gut dysbiosis elevated LPS and disrupts key metabolites like SCFAs, reducing axonogenesis‐related gene expression and impairing thalamocortical and thalamic axon development in offspring [5]. Nevertheless, the mechanistic link between SCFA levels and their impact on offspring cognitive function and synaptic plasticity remains poorly understood. Gut dysbiosis elevates LPS levels, which impair fetal neurogenesis via placental inflammation through NF‐κB and IL‐6 signalling, leading to neural tube closure failure [6]. Furthermore, research has demonstrated that butyrate functions as an inhibitor of histone deacetylase (HDAC) activity, thereby modulating the epigenome through chromatin remodelling mechanisms [7]. The above changes have profound effects on neurodevelopment by activating inflammatory pathways via the brain‐gut axis, modulating gene expression, and interfering with epigenetic mechanisms in the early embryo.

Maternal gut dysbiosis during pregnancy can lead to a reduction in SCFA and 5‐HT, which increase osteoclast activity and inhibit osteoblast activity, thereby impairing fetal bone formation [8]. An early chicken model demonstrated that LPS‐induced ROS accumulation and upregulation of antioxidant genes enhance Sox9 transcriptional activity, which in turn downregulates key downstream targets like Runx2, ultimately disrupting normal cartilage formation in the fetus [9]. Elevated LPS also leads to the upregulation of retinoic acid receptor α (RARα) and the downregulation of the expression of the osteogenic gene DLX5, ultimately restricting the development of long bones [10]. Although existing studies have shown that maternal dysbiosis can affect embryonic skeletal muscle development through elevated LPS and decreased SCFAs and 5‐HTHowever, these findings are mainly based on animal models and lack direct evidence to validate their mechanisms in humans.

Maternal gut dysbiosis during pregnancy disrupts fetal microbiota colonisation and impairs the permeability and intestinal barrier integrity [11]. Mouse experiments show that maternal dysbiosis leads to elevated LPS levels. This results in a reduction of innate immune components and an increase in inflammatory markers, which in turn causes a higher prevalence of gastrointestinal diseases in the fetus, such as NEC. Furthermore, a decrease in maternal *Limosilactobacillus reuteri* during pregnancy alters propionic acid levels, disrupting the GPR41–GDNF/RET/SOX10 signalling pathway and impairing the development of the enteric nervous system (ENS) in the mouse offspring. Although current evidence suggests that maternal gut homeostasis plays a crucial role, the signalling pathways mediated by microbial metabolites remain poorly understood. Elucidating these mechanisms is essential for understanding the developmental programming of the gastrointestinal system in early life. In light of the gut microbiota's role in aging, investigating how maternal gut microbiota might influence offspring aging via its impact on establishing the offspring's gut microbiota is a promising area for future exploration.

Fetal kidney development is typically completed between 32 and 36 weeks of gestation. Maternal gut microbiome dysbiosis during pregnancy can lead to abnormal development of the fetal urinary system, including congenital kidney abnormalities, urinary tract abnormalities (CAKUT), and kidney dysfunction [12]. Elevated maternal IS during pregnancy promotes dysbiosis, causing increased inflammation. This, in turn, affects the proliferation and differentiation of fetal kidney precursor cells, impacting the formation of renal tubules and glomeruli. Moreover, maternal supplementation of fatty acids during pregnancy may be associated with fetal CAKUT, particularly the development of cystic kidneys. Although current research on the influence of maternal gut microbiota on embryonic urinary system development is relatively limited, existing studies suggest that elevated IS levels can affect the proliferation and differentiation of embryonic nephron progenitor cells by activating inflammatory and oxidative stress pathways. However, it remains unclear whether the gut microbiota interferes with the formation of fetal nephrons through a metabolite‐inflammation axis. Future studies should further focus on whether supplementation with probiotics such as SCFAs could serve as a new strategy to reduce fetal urinary system disorders.

The respiratory system of mammals originates from the ventral wall. Animal models demonstrated that increased LPS derived from maternal dysbiosis can induce fetal lung cell developmental disruption through oxidative stress and NF‐κB signalling [13]. Supplementation with *Lactobacillus johnsonii* during pregnancy can alleviate lung injury caused by bronchopulmonary dysplasia in neonatal mice. Nevertheless, the underlying mechanisms remain unclear. There is still a lack of systematic mechanistic studies on whether gut flora and their metabolites are directly or indirectly involved in the differentiation of lung progenitor cells and whether they affect classical developmental signalling pathways such as SHH and Wnt during the early embryonic stages. Solving this problem will help to reveal the precise regulatory mechanisms by which maternal microecology influences embryonic lung development.

The development of the embryonic/fetal reproductive system is an intricate process. Maternal exposure to dibutyl phthalate (DBP) during pregnancy causes dysbiosis, characterised by an increase in the relative abundance of *Bacteroides*, *Prevotella*, and *Prevotellaceae*, which can affect testicular damage in the offspring. The genital ridge cells (GRCs) originate from the mesoderm during the gastrulation stage and are regulated by classical signalling pathways such as Wnt and BMP [14]. Microbial metabolite Equol regulates GRCs differentiation via ERβ. Epigenetic reprogramming in mammals occurs in primordial germ cells. It is worth further investigating whether maternal dysbiosis may mediate transgenerational inheritance by affecting the migration and epigenetic reprogramming of GRCs.

Maternal gut microbiome dysbiosis during pregnancy disrupts the development of the fetal immune system [15]. Metabolites produced by the maternal gut microbiota, such as SCFAs, BAs, and LPS, can cross the placenta and influence fetal energy metabolism and inflammatory status. Especially, drastic changes in SCFAs may impair fetal metabolic reprogramming [16]. However, most of these studies have focused on inflammation induced by changes in microbial metabolites, while direct evidence is still lacking regarding whether the gut microbiota and its metabolites contribute to the development of metabolic and immune‐related disorders by modulating epigenetic programming during embryogenesis. Future research should focus on how the maternal gut microbiota influences determinants and potential intervention windows for the development of metabolic and immune‐related diseases (Figure 1).

**FIGURE 1:**
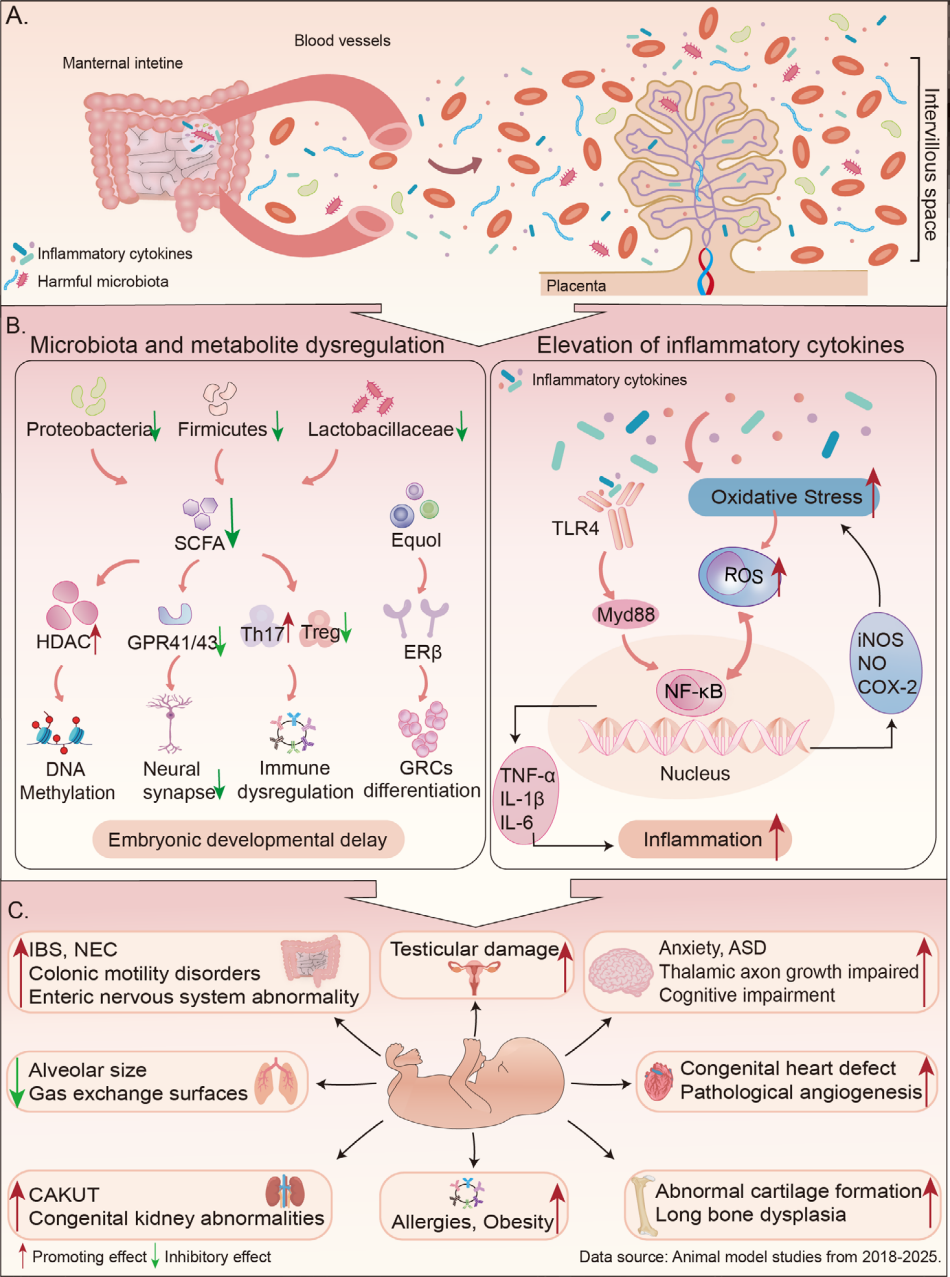
The impact of maternal dysbiosis on embryo/fetal development. (A) Maternal dysbiosis: Maternal intestinal dysbiosis can be transmitted vertically to the embryo/fetal through the placenta. (B) Transmission mechanisms: Maternal dysbiosis leads to a reduction in *Proteobacteria*, *Firmicutes*, and *Lactobacillaceae*, resulting in decreased SCFA production. A decrease in SCFA primarily alters epigenetic regulation by affecting HDAC activity, thereby influencing gene expression. It also impairs synaptic development by reducing the activation of GPR41/43 (the direct effect of microbial metabolites on DNA methylation still requires in vivo validation). SCFA deficiency suppresses anti‐inflammatory Treg cells and promotes the activation of pro‐inflammatory Th17 cells. Additionally, the microbial metabolite Equol regulates genital ridge cell differentiation via ERβ. Ultimately, this leads to disruption of the embryonic immune microenvironment and delayed embryonic development. In addition, maternal gut microbiota dysbiosis can lead to elevated levels of inflammatory cytokines, activation of the NF‐κB signalling pathway and induction of oxidative stress. Among them, NF‐κB and ROS generation have a mutually promoting relationship, affecting cell survival, migration, and differentiation during embryonic/fetal development. (C) Affect fetus development: When the maternal flora spreads to the embryo/fetal, it can cause abnormalities in the growth and development of the embryo/fetal, affecting various systems such as the nervous system, cardiovascular system, musculoskeletal system, gastrointestinal system, urinary system, respiratory system, reproductive system, and other immune systems.

Maternal exposure to adverse environments or disease conditions during pregnancy is closely associated with gut microbiota dysbiosis, which may lead to reduced microbial diversity and an increase in pathogenic bacteria. Mutually, gut microbiota dysbiosis can further exacerbate the onset and progression of diseases. Maternal dysbiosis‐induced embryonic/fetal dysplasia is frequently linked to an inflammatory condition and an excessively heightened state of oxidative stress. Therefore, for pregnant women with diseases or exposed to adverse environments during pregnancy, it is crucial to pay attention to the impact of gut microbiota on the embryo/fetus. While recent studies have begun to elucidate specific molecular mechanisms through which certain gut microbiota influence embryonic development, the role of the maternal gut microbiome is gaining increasing attention, particularly in regulating embryonic DNA methylation. Distinct from previous work, our study specifically addresses how maternal gut microbiota dysbiosis impacts fetal development. We systematically investigate how gut microbiota‐derived metabolites, such as LPS and SCFAs, modulate inflammatory responses, oxidative stress, and key developmental signalling pathways, thereby influencing embryonic outcomes.

Extensive interactions exist between NF‐κB, ROS, and classical developmental signalling pathways. These proteins that maintain cellular homeostasis often also exhibit regulatory roles in cell fate determination and differentiation. Embryonic development exhibits spatiotemporal specificity. Thus, both the timing of maternal gut microbiota dysbiosis during pregnancy and the cell type–specific sensitivities to microbial signals and distinct metabolites may underlie the variability in embryonic developmental outcomes. For example, recent studies have found that the maternal gut bacterium *Akkermansia muciniphila* has the ability to shape neural and intestinal stem cells in the offspring, and that SCFAs and amino acid metabolites can influence stem cells in the offspring by modulating the mTOR signalling pathway [17]. However, current research in this area remains very limited. It is worth noting that current research primarily relies on mice and other animal models, but the gut microbiota of animals differs from that of humans, and the environmental and microbial exposure experienced by animals in daily life is significantly lower than that of humans. Moreover, the composition and function of the gut microbiota vary significantly between individuals, closely related to genetic background, dietary habits, and living environment [18]. Therefore, significant challenges remain in translating these findings into clinical applications. However, animal experiments no doubt could provide a basis for the molecular mechanisms underlying future clinical translation.

With the advancement of high‐throughput sequencing technologies and artificial intelligence (AI), the scope of microbiome research is gradually expanding. Currently, metagenomic sequencing is being used to detect changes in the composition and function of the microbiota during pregnancy. Machine learning (ML) and deep learning (DL) have been used to identify and predict microbiomes under different health conditions [19]. Additionally, AI tools can be used to simulate the infant microbiome and explore how the gut microbiome influences infant development. Early microbiota monitoring and dietary interventions in pregnant women may help mitigate adverse effects on embryonic and fetal development [20]. Current studies have shown that probiotic supplementation during pregnancy can help improve adverse pregnancy outcomes. For example, high‐risk pregnant women who consumed probiotics containing *Lactobacillus* exhibited a reduction in systemic inflammation. However, there is currently no standardised definition or classification for the degree of gut microbiota dysbiosis during pregnancy. Therefore, further research is needed to elucidate the functional mechanisms of key maternal gut microbiota and their metabolites during pregnancy and to establish a standardised risk stratification system. Ultimately, the goal is to protect embryonic and fetal development by regulating the maternal gut microbiota within the maternal‐fetal environment.

## Author Contributions

N.F. and S.M. collected cardiovascular, nervous system, and musculoskeletal data; Y.S. and G.W. collected digestive and other system data; N.F. and Y.S. designed charts and integrated mechanisms; G.W. and X.Y. revised discussion and translational sections.

## Ethics Statement

Cited studies comply with ARRIVE guidelines.

## Conflicts of Interest

The authors declare no conflicts of interest.

## Data Availability

Data sharing not applicable to this article as no datasets were generated or analysed during the current study.
